# Promoting Generalized Learning in Balance Recovery Interventions

**DOI:** 10.3390/brainsci11030402

**Published:** 2021-03-22

**Authors:** Sara A. Harper, Anne Z. Beethe, Christopher J. Dakin, David A. E. Bolton

**Affiliations:** 1 Department of Kinesiology and Health Science, Utah State University, Logan, UT 84322, USA; sara.harper@usu.edu (S.A.H.); anne.beethe@usu.edu (A.Z.B.); chris.dakin@usu.edu (C.J.D.); 2 Sorenson Legacy Foundation Center for Clinical Excellence, Utah State University, Logan, UT 84322, USA

**Keywords:** postural perturbations, balance reaction, falls, perturbation-based balance training

## Abstract

Recent studies have shown balance recovery can be enhanced via task-specific training, referred to as perturbation-based balance training (PBT). These interventions rely on principles of motor learning where repeated exposure to task-relevant postural perturbations results in more effective compensatory balance responses. Evidence indicates that compensatory responses trained using PBT can be retained for many months and can lead to a reduction in falls in community-dwelling older adults. A notable shortcoming with PBT is that it does not transfer well to similar but contextually different scenarios (e.g., falling sideways versus a forward trip). Given that it is not feasible to train all conditions in which someone could fall, this limited transfer presents a conundrum; namely, how do we best use PBT to appropriately equip people to deal with the enormous variety of fall-inducing scenarios encountered in daily life? In this perspective article, we draw from fields of research that explore how general learning can be promoted. From this, we propose a series of methods, gleaned from parallel streams of research, to inform and hopefully optimize this emerging field where people receive training to specifically improve their balance reactions.

## 1. Introduction

Falls are a leading cause of injury among older adults in the United States [1], and given their great economic, health, and societal costs [2], there is a need for effective fall prevention strategies. Many age-related factors, such as decreased lower body muscular strength, functional mobility, sensory acuity, and cognition, contribute to increased fall risk [3,4,5]. Regardless of the cause, once balance is lost, the body’s last line of defense to prevent a fall is a compensatory reaction, often in the form of a step or a reach-to-grasp to regain balance [6,7,8]. Thus, training compensatory balance reactions could be an important way to reduce fall risk in vulnerable populations. Accumulating evidence indicates that these reactions can indeed be improved through practice, like other voluntary motor skills [9,10,11,12,13], by repeatedly disturbing posture through external perturbation (e.g., waist pull, sliding floor, etc.), a process known as perturbation-based balance training (PBT).

Although PBT improves balance reactions and resistance to falls, training gains are largely limited to the condition that was specifically practiced (e.g., [14,15]). This can be problematic given the wide assortment of fall scenarios we face in daily life. As we discuss later, one potential means to improve the generalization of balance recovery skill is to expose people to a variety of training conditions and emphasize the need for behavioral flexibility. Our aim in this perspective paper is to first outline the strengths and limitations of current balance reaction training systems and then to propose how to build upon these systems to improve the effectiveness of PBT beyond what is currently observed.

## 2. Current Approaches to Training Balance Recovery Skills

Several interventions, including traditional resistance exercise and balance training (e.g., voluntary weight shifts, standing with a reduced support base, etc.) have been used to reduce fall risk [16,17,18,19,20,21,22], (e.g., effect sizes of 0.74 [16], and 0.71 [17], for multiple component interventions; 0.79 [18] and 0.63 [23] for exercise interventions). Although beneficial, it has been noted that these methods yield only a modest reduction in fall frequency [24], which has prompted some researchers to advocate for interventions that more effectively reduce fall risk in vulnerable groups [11,24,25]. One such approach is to train people’s compensatory reactions directly.

When balance is lost, individuals have only a short period of time to coordinate and stabilize the body [19]. The ballistic nature of these compensatory reactions differs from the slow, isolated actions that characterize many resistance exercise programs used in clinical settings. Moreover, exercises in the clinic are often predictable and are presented with limited variety (e.g., repeated leg and hip extension actions in a seated leg press). In contrast, postural disturbances in naturalistic settings vary greatly, and tend to be highly unpredictable [25]. This discrepancy between the slower and more predictable types of training performed in the clinic versus the ballistic and unpredictable reactions that occur in the real world appears to contribute to the limited applicability of such training [24]. To overcome this limitation, recent PBT studies have employed methods such as treadmill accelerations and/or decelerations, moveable platforms that shift or tilt [26,27,28,29,30], and therapist-induced nudges or pulls that better approximate real-world disturbances. While the PBT approach is closer to the conditions observed during falls in daily life, there remains room for improvement. For example, most PBT studies have relied exclusively on backward slips or forward trips, in part due to the greater accessibility (cost, availability, and space requirement) of equipment used to apply perturbations in the anterior-posterior plane, such as a treadmill. The limited variety of postural disturbance, as well as the use of simple environments, may reduce the generalizability of adaptations to PBT training [31]. Despite such limitations, there is strong evidence that PBT training can enhance fall resistance and thus provide an important foundation for further development as we discuss next.

### 2.1. Evidence for the Effectiveness of PBT

Current research suggests that compensatory balance reactions can be developed using PBT [9,10,11,32] and that these improvements can be sustained for up to a full year following training [30]. Meta-analyses have shown PBT to be effective at reducing falls (both in the laboratory and the community) in healthy older adults and in individuals with Parkinson’s disease [9,10] (PBT intervention effect size = 0.54 [9], volitional and reactive stepping intervention effect size = 0.48 [10]). In the laboratory, PBT has led to tangible improvements in participants’ compensatory balance responses (e.g., increased stability revealed by the relationship between center of mass and base of support using video analysis [27]), in addition to a reduction in the frequency of ‘falls’, which in the laboratory are typically classified as load acceptance into a safety harness. Such findings indicate that PBT is an effective way to reduce falls in the laboratory and therefore has the potential to benefit daily life. Recently, when PBT was compared to a multimodal exercise-based balance training control group (i.e., the ‘standard care’ group), PBT led to greater improvements in fall prevention versus standard care [33]. As might be expected, the retention of balance training adaptations observed in older adults is less than in healthy young adults [15]. Nevertheless, it is encouraging that balance reactions in older demographics can indeed be improved with training.

A variety of PBT methods have been used successfully to train people’s compensatory balance reactions to reduce the likelihood of falls. A well-studied model is the use of slip perturbations induced by a moveable platform embedded in a walkway [26,28,29,30,34]. Collectively, studies using this approach reveal improved slip resistance with practice, and these improvements were retained days and even months after training. After a single session, participants produced more effective compensatory reactions (i.e., they showed better stabilization of the center of mass in terms of position and velocity) and fell less often. Although training effects measured within the same session could theoretically be attributed to factors other than learning per se (such as arousal), the sustained improvements observed in follow-up tests months later provide strong evidence that these changes were indeed learning related. The retention observed in older adults following slip training (up to a full year after training took place) is also remarkable given that this occurred after only a single session of 24 slips [30]. Together these results indicate that PBT not only works, but it is also highly efficient. Perhaps of greater value, however, is the fact that the benefits of slip training can extend beyond the laboratory and have been associated with a reduction in real-life falls in community-dwelling older adults [28]. Up until this point we have highlighted slip training studies that demonstrate positive outcomes associated with PBT in its most constrained form—i.e., a single type of perturbation trained in a single session. We will now examine some of the limitations of such PBT.

### 2.2. Limited Transfer between Conditions

Although PBT improves balance reactions and resistance to falls, its benefits are limited largely to the specific conditions experienced during training. For instance, two days of anterior-posterior perturbations in standing participants does not improve compensatory balance responses to untrained medial-lateral perturbations for which the participant has not received training [14]. This lack of generalization for people with an untrained perturbation direction is important for two reasons. First, lateral instability is particularly challenging for older adults [35], and yet, most PBT studies focus on perturbations acting in the sagittal plane (e.g., the use of treadmill acceleration/decelerations [36,37,38,39,40], but also forward sliding platforms [30,34]). This suggests that many current PBT approaches may not improve balance recovery along the vulnerable frontal plane. Second, and more broadly speaking, these findings show that the improvements garnered by training balance reactions in a specific direction may not transfer to similar reactions in other directions [11]. It seems that directional specificity, both in terms of perception of the event and the ensuing motor response, is essential to develop balance recovery skill and that directional variety during training may therefore be important for generalization [24].

Transfer of motor skill in people from a trained condition to an untrained one has been demonstrated following PBT, but only to a limited degree. For example, slip training on a sliding platform leads to a more effective compensatory response when slipping on an oily surface for the first time [27], or while walking on a treadmill [34]. Likewise, interlimb transfer has been observed when an untrained leg is suddenly exposed to a slip that was previously only experienced in the contralateral leg [26]. In both cases, the high degree of similarity between the two slips experienced and their compensatory response is an example of ‘near’ transfer, which is to say the training situation is similar to the ‘test’ situation, where learning is evaluated. In addition, while slip training in the laboratory has been shown to lead to fewer falls in daily life [28], it is unclear whether this slip training prepares people to compensate for different types of perturbations, or only those where a slip reaction is appropriate. To illustrate, Rosenblatt et al. [41] observed fewer trip-related falls in older adults following several sessions of treadmill trip-perturbation training (i.e., unexpectedly decelerating the treadmill when establishing a stance leg). Unfortunately, this treadmill trip-perturbation training did not translate to a change in the number of ‘all-cause’ falls, suggesting that only the ‘trained’ trip response improved and therefore only falls requiring this response would benefit from such training. Similarly, training a trip reaction on a treadmill, which requires a compensatory step, does not appear to improve compensatory step responses during a lean and release task [15]. In this latter case, the lack of transfer is striking given that both tasks require detection of a forward fall and a quick step forward to establish a new base of support. One logical conclusion to draw from this body of research is that training across a variety of perturbation types and directions is needed to develop a broader capacity for balance recovery.

To develop a ‘general’ capacity to resist the most common causes of a fall, PBT may require exposure to a wider sample of the most likely postural disturbances experienced in daily life. Such training could be complemented with practice identifying and switching between effective responses to suit a given context. The current emphasis of PBT studies using one, or perhaps two, types of perturbation is a sensible approach in some regards, because it allows for precise experimental control and enhances improvements in the performance of the specific skill trained. However, this specificity comes at the cost of generalizability. Exposure to a broader sample of postural disturbances would slow learning, and introduce additional confounding variables, but would also likely improve compensatory responses to a wider range of disturbances, as well as improve the capacity to switch between compensation tactics. Training in this manner could be important in scenarios where a highly automatic reaction needs to be suppressed and a new action selected to prevent a fall [7,13,37]. Such behavioral flexibility requires the cognitive ability to recognize problems, suppress the current response, select an alternative response, and implement it. It is interesting to note that measures of behavioral flexibility and inhibitory control have each been shown to predict falls in community-dwelling older adults [42,43]. Although the specific mechanisms by which these cognitive constructs contribute to fall prevention are unclear, these findings are at least suggestive that behavioral flexibility plays an important role in fall prevention. Indeed, behavioral flexibility itself may be a logical intervention target to compensate for global reductions in physiological capacity (e.g., impaired balance) [42].

Although PBT has proven effective, development and optimization of this intervention is still in its infancy. In the next section, we discuss how balance recovery skills could be developed in a more abstract manner where a set response is not linked to a specific perturbation. Research outside the domain of balance control offers insight into what promotes generalization of learning, and while application to how we train corrective balance is speculative at present, this information could help guide future efforts to improve generalizability of PBT.

## 3. Can We Train Peoples’ Behavioral Flexibility to Preserve Upright Posture?

### 3.1. The Importance of Transfer of Learning

The ultimate goal of most classroom, laboratory, or clinical training programs is that the skills developed during training will transfer to the untrained situations that we confront in daily life. To meet this goal, training methods that improve performance and generalize beyond peoples’ trained context must be identified [44]. Even though current research suggests that transfer across tasks is uncommon, several authors have reported that the reason for this may be that experimental conditions are, either accidentally or by design, biased towards developing specific skills [44,45,46,47]. Many studies use training methods that fail to encourage transfer and as will be discussed later, could be remediated by using training methods that develop the cognitive processes that contribute to the behavioral flexibility necessary to generalize across tasks. As a common theme, training in more complex and variable environments appears necessary for learning that results in transfer [44,46,48,49]. In the next section, we briefly review evidence for the conditions necessary for transfer.

### 3.2. What Are the Necessary Conditions for Transfer?

Generalization of learning is fundamental to a variety of fields such as education, athletics, and rehabilitation. Not surprisingly, the topic of generalization has sparked a great deal of interest and research over many decades [45,47], and this could be used to inform PBT. A theme underlying the emergence of generalization is the introduction of sufficient variability, complexity, and cognitive engagement. For example, athletes’ skill often transfers beyond their sport of specialization. Expert athletes in several sports (basketball, volleyball, and water polo) exhibit superior performance on a variety of cognitive and perceptual measures, such as response speed, selective attention, and spatial orientation ability, when compared with novices [50]. Expert baseball players’ performance on a classic test of inhibitory control—a go/no-go task—is also significantly better than novices, suggesting baseball training may lead to improved inhibitory control [51]. One key difference between athletic training and PBT is that in addition to training of specific skills, sports involvement includes the training and application of these skills in extremely variable, complex, and unpredictable environments. It should be noted, however, that individuals already gifted in these mental skills may have self-selected into these sports, and therefore caution must be taken in concluding that the sports training itself is the sole cause of their exceptional ability.

Similar to athletics, video game play also provides clues into what is necessary for generalization of learning. Although much work in this field suggests that video game expertise is largely restricted to the game trained upon, an interesting exception has been observed for action (i.e., first-person shooter) video games [46]. Experienced action gamers show superior performance compared with non-gamers in a variety of cognitive tests that emphasize abilities including multi-tasking/task switching [52,53,54], visual spatial cognition [55], and attentional control [56,57]. Compared to many other video games, first-person shooter games require quick decision-making in variable, complex, and unpredictable environments. The constant demand for attention and cognitive flexibility in these games has been argued to be a key mechanism for promoting transfer of skill beyond the games themselves. In this case, it appears that the cognitive processes being trained are broadly applicable to a range of other tasks. This idea is consistent with the ‘transfer-appropriate processing theory’, which posits that the major driving force behind positive transfer is a similarity in cognitive processes between the trained task and the transfer task [49]. According to this theory, tasks do not need to share similar motor elements per se, but instead need to share cognitive processing demands such as an emphasis on rapid decision-making, multi-tasking, or control over attention.

A recent meta-analysis comparing video gamers versus non-gamers provides support for the link between action video game play and superior cognitive performance [48] (Hedges effect size g = 0.358 for video game interventions). What was particularly compelling was that this relationship was preserved in the most rigorous studies in which naïve subjects were randomly assigned to train either on an action video game or a control game. This type of research design eliminates potential issues with selection bias whereby people that were already better at certain cognitive abilities may have taken up active gaming in the first place, as opposed to developing such skills through the games themselves. It also controls for motivation effects. By engaging both groups in challenging, yet enjoyable games, arousal is enhanced, and learning facilitated. In one of these studies, for example, a group was trained on a first-person shooter game (Medal of Honor: Allied Assault, Electronic Arts, Redwood, CA, USA), while a second control group was trained on Tetris, which was previously shown to only lead to game-specific improvements [58]. In the end, the first-person shooter video game group demonstrated significant improvements in visual attention performance compared to the control group. Similarly, when naïve subjects were trained on an action video game (Unreal Tournament, Atari, Sunnyvale, CA, USA), they demonstrated superior visual contrast sensitivity when compared with a control group that was trained on a non-action video game (The Sims 2, Electronic Arts, Redwood, CA, USA) [59].

### 3.3. Relationship to Training Balance Recovery Skills

A common theme across both athletic and first-person shooter training regimens, as well as other studies which show transfer to untrained tasks [44], is that they are extremely complex and engage numerous cognitive systems. This differs from the types of tasks often used in laboratory settings, which are motorically simple, and where discrete domains of cognitive ability (e.g., inhibition versus working memory) tend to be purposely separated out for greater experimental control [44]. Since compensatory reactions can be trained in people similarly to other voluntary motor skills [9,10,11,32], it seems logical that the principles that contribute to transfer can also be applied to train peoples’ compensatory balance reactions. It is our view that a key ingredient missing in training peoples’ capacity for effective balance recovery is the imposition of variety and complexity. Exposure to these variables has two aims, first to expose trainees to a more representative sample of the postural disturbances and possible responses that they would experience in daily life (variety) and second to enhance cognitive involvement to aid generalization of such training (complexity). The variety of disturbances should include and cover the most common postural disturbances for a particular group with high fall risk or consequence (note: Ideally, this would be drawn from the natural statistics of falls in this group). Similarly, trainees should also be exposed to the most common possible compensatory responses to these disturbances, which encompasses some of the complex settings where balance recovery needs to happen. This suggestion draws from the concept of ‘statistical overfitting’ whereby exposure to a limited sample can limit generalization [60]. However, because training all possible scenarios is unfeasible, as will be discussed below, future research should identify the minimum number of scenarios that provides generalizability across the most common scenarios. To further promote generalization, cognitive processes, such as attention, should also be stressed [49], not only as a source of interference (such as with dual-task training), but as an intrinsic part of the recovery process [61]. Specifically, attention needs to be directed towards quickly identifying the postural challenge and generating the most appropriate response, as would occur during a natural fall. To foster generalization, we want to uncouple rigid stimulus-response relationships and facilitate the flexible selection of responses suitable for the available environmental affordances and constraints on action.

### 3.4. Summary

With the ultimate goal to develop skills that transfer to real-world scenarios, consideration should be given to the variability, complexity, and cognitive challenge of the training environment. Athletes’ and video gamers’ performance across a range of measures provides evidence for improved transfer to untrained tasks, and this appears to be due to their highly variable and complex training environments. Extending from this work, we suggest that when training balance recovery in people, the training environment needs to be variable and complex to facilitate transfer and to foster general learning. This would also include practice switching between the most common compensatory responses, and postural disturbances to prepare people for the unpredictable nature of real-world falls and to allow them to call upon the appropriate response when the time comes.

In the next section, we offer some potential ideas for clinical application and future study. Note, several of these points borrow from and/or build upon successful ideas that others have presented. It is important to recognize that we are not attempting to provide an exhaustive overview of the exact ways in which we can increase variability and complexity to drive cognitive involvement. Instead, we offer a basic framework that could help shape clinical practice. Our main goal is to highlight that increasing task variability and cognitive demand through complexity during PBT may lead to better generalization of balance recovery skills.

## 4. Recommendations for Clinical Application

### 4.1. Relationship to Training Balance Recovery Skills

The high economic and personal costs associated with falls [1,2] makes it worth the investment to seek methods which optimize PBT and ultimately reduce fall rates in vulnerable groups. Indeed, the significant benefits accrued from PBT in a short time frame with minimal practice [30] suggest it serves as a strong foundation on which to build upon. It also seems logical to combine traditional forms of resistance and aerobic training with PBT as part of a comprehensive regimen since PBT offers direct training of balance reactions [24], while standard exercise offers broad health and wellness benefits [62,63,64] in addition to bestowing some resistance to falls [24].

A gradual increase in challenge is a necessary feature of training programs designed to foster skill adaptation [49]. In PBT, the notion of a ‘challenge’ can be framed in several ways. This includes manipulating training factors such as (a) the parameters of the perturbation, (b) the complexity of the environment in which responses must be made, and (c) multi-tasking (both motor and cognitive) [33,65,66]. From these factors, the challenge can be increased, for example, by gradually imposing a larger perturbation over time. In this case, one could imagine graduating from a small perturbation to a more forceful perturbation, where the former is manageable via a feet-in-place reaction and the latter requires a change of support to prevent a fall [7,25]. Alternatively, the direction and/or predictability of the perturbation could be adjusted [66]. Beyond the issue of how the perturbation is delivered, features of the environment can be altered to make conditions more or less difficult to regain balance. For example, by introducing obstacles to avoid, or targets for balance recovery (e.g., stable handle), movement options can be constrained or afforded, forcing the involvement of higher-level decision-making [67,68,69]. Indeed, even a person’s state of motion (e.g., walking or transitioning from sitting to standing) at the time a perturbation is delivered can be manipulated. To illustrate, forcing someone to walk faster than their preferred pace can challenge their capacity to recover balance in the event of a postural disturbance [70]. Lastly, the introduction of a secondary cognitive task [66], distracting sensory stimuli [71], reduced lighting [72], or concurrent engagement in a separate motor task (e.g., carrying groceries) [73], are all factors that influence gait and posture, and can all be used to increase task-specific transfer [74]. It is notable that such manipulations also tend to amplify cognitive burden [66,75]. In broad terms, when we discuss the idea of task variation in PBT, it could involve introducing any of these elements alone or in combination. The main idea is to challenge the system with sufficient variability to force the development of cognitive flexibility [44,46].

Similar to any motor skill training program, there are key elements that contribute to outcome success. The initial level of PBT challenge should be tailored to the individual through baseline assessment, and then challenge of the task can be adjusted to keep pace with an individual’s progress [33,65,66]. To maximize participant adherence and engagement, training must be challenging, but also motivating and enjoyable [76,77,78,79]. The ultimate goal of a complete PBT program should be to arm individuals with a sufficient repertoire of skills to allow them to manage the wide variety of scenarios in which falls may occur in daily life.

### 4.2. Points to Consider When Designing PBT Training Protocols for Fall Prevention

1. Individualization of training.
  - Individualized baseline. The baseline assessment is a critical stage to determine the specific needs for a given individual [9,80,81,82]. In some cases, training may need to start very simple to develop a component skill in isolation before advancing to a more difficult task (e.g., practicing a step response when nudged by a therapist).
  - Personalized progression. Training increments need to cater to the individual. The idea is to progressively increase demand based on the individuals’ stage of skill development, and always within a safe setting (e.g., catch harness). We suggest initial training blocks that focus on a component skill (e.g., train slip on one day, then trip another day) before moving on to a random mixture, and only after some mastery of each skill is achieved [24,31]. Again, these increments need to be guided by carefully monitoring the patient’s ability instead of relying on arbitrary set points.
2. Perturbation parameters.
  - Predictability. In the early stages, it is okay if the individual is aware of the perturbation details (intensity, direction, location, etc.), but this should change as the training program is made more difficult over time. Changing predictability is important because people rarely have advanced knowledge of the event that causes the loss of stability, and foreknowledge of the event gives a clear (and unnatural) advantage to fall prevention mechanisms [83]. To provide the most realistic training, the goal is to develop the reactive component of balance recovery rather than anticipatory mechanisms.
  - Direction specificity. Transfer of balance recovery skill to an untrained condition is limited. Thus, an optimal program needs to eventually expose people to various perturbation directions, to imitate real life where a loss of balance can occur in any direction. A simple and reasonable method to enhance PBT training is to gradually progress towards including a range of perturbation directions [74]. Depending on baseline characteristics, it would be prudent to start with a single direction of perturbation, before practicing a new direction on a later date. Training could start with a slip, for example, then progress to a trip on separate days. Lateral stepping and compensatory upper limb responses should also be trained subsequently. PBT could progress to involve a random mixture of perturbation directions, which could span the range of all likely perturbation directions in the latter stages of training.
  - Intensity. Start with manageable, smaller perturbations and then progress to larger ones. Intensity changes may also include changing the speed the participant is walking when the perturbation occurs. Given that we tend to scale our reactions to what we expect, intensity should eventually be randomized and unpredictable. For examples of recent studies where walking speed and perturbation intensity in balance training are individually adjusted, see [66,84].
3. Response environment. The environment under which a fall occurs generally places several constraints on the appropriate compensatory responses, where some responses will have a higher likelihood of success. As the training program progresses, the constraints and responses afforded should be manipulated along with variables which impair or challenge the response decision process, exemplified by changes in lighting or presence of distracting stimuli. While perturbation parameters are sometimes manipulated in standard PBT (e.g., randomized slips and trips whilst walking [38,39]), adjustments to the response environment are much less common and may enhance skill transfer through the introduction of task variation [74,85]. Some options to consider are:
  - Imposing an obstacle to a step or manipulating the presence of a support handle. Introducing options and restrictions on balance recovery could amplify the demand on behavioral flexibility and encourage participants to learn more flexible ways of responding to a loss of balance.
  - Altering the types of obstacles and handles may provide a unique opportunity to instill deeper learning of how to establish a new support base without relying on any particular cue or afforded response. For example, a standard safety handle could be presented in one case, but then a flat countertop in another. The idea is to learn how to adapt the upper limb response to whatever support base is available (i.e., one involves grasping a handle, while the other involves planting a flat hand to brace the body). The premise here is for patients to learn abstract procedures for how to recover balance using whatever effectors are available and not to become dependent on a specific sensory cue.
  - Challenging the decision process by manipulating information processing. For example, training to react under a dim lighting condition where environmental cues are harder to recognize or dealing with random sensory distractions (e.g., sound or lights) could be useful to mimic some of the settings we face in daily life, such as when we walk down a busy city street.
4. Cognitive Challenge. Contending with complex situations involves greater reliance on cognitive resources. The idea of adjusting cognitive challenge overlaps with some of the points already presented, such as adding complexity to the response setting, adjusting the predictability of the perturbation, or dealing with distraction. That said, manipulating cognitive challenge is worth exploring independently given the distinct training needs for dual-tasking versus direct cognitive involvement in the balance recovery task.
  - Dual-task training involves performance of a cognitive task (e.g., counting backwards by serial sevens) in parallel with the balance recovery task. In this way, the cognitive task is a source of interference or at least is in competition for the same cognitive resources. Recent PBT programs have begun adding this element to their training regimen [66], informed by the knowledge that there is degraded performance in cognitive and postural tasks when both run concurrently [86]. The idea is that we can train our capacity to manage both tasks and are therefore better equipped to resist a fall if we lose balance while engaged in a separate cognitive task.
  - Cognitive abilities must often be directly involved in solving the challenge of recovering balance. Here, we draw attention to the fact that the ability to dual-task and the direct use of cognitive resources to recover balance are both important in everyday life, but the latter is often neglected. This could be a significant oversight given that increasing complexity in adapting to the task at hand (i.e., solving the loss of balance problem) would help develop generalization in balance recovery skills. Using higher brain processes to adapt our reactions to a complex setting is distinct from dual-task training where attention is diverted away from the balance recovery task. The benefits of directly engaging cognitive resources to aid skill acquisition versus dual-tasking has been recently explored (See [61]).
  - Aside from dual-tasking where a cognitive task is managed concurrently with the balance task, we also should consider engagement in a simultaneous motor task such as carrying groceries while walking. Such tasks present a challenge in allocating attention, which may be a trainable skill—one that could be introduced as a participant improves over the course of training. It is notable that disengaging from an ongoing motor task, such as holding onto an object, can delay onset in the perturbation-evoked compensatory response [73]. Thus, training to overcome this type of challenge could represent another important consideration when seeking to optimize PBT.
  - Contextual interference. Perhaps counterintuitively, evidence suggests that the in-depth cognitive processes required to contend with learning several tasks concurrently (i.e., contextual interference) can result in more robust learning [74,85]. In the case of PBT, multi-directional training is an example for how task complexity could be increased to introduce contextual interference. Notably, this idea of interference could equally involve manipulation of other parameters discussed above.
5. Perturbations during gait and/or during transitions. To have the greatest relevance to fall prevention in daily life, training during gait or in-motion states is essential [70]. Due to the extra complexity, this skill should be added further along in the training progression and is dependent on the peoples’ starting point. While PBT during walking has been completed, many studies will use gait speeds or perturbation intensities that are set in absolute terms (e.g., 3 mph) and not per individual (e.g., 0.8 times the speed of the individual’s self-selected walking speed).
6. Keep motivation/arousal optimal to facilitate learning. If the skills trained are too simple or difficult, optimal learning will be curtailed. Therefore, programs need to be individualized to keep the challenge appropriate for each individual, and the program should be planned and implemented in a way that makes it fun and ‘time well spent’. This will encourage participant engagement to promote learning. In addition, if numerous training sessions will be required over the course of several weeks, adherence must be ensured for success. One way to potentially create a fun learning environment while gradually increasing the challenge is to use gaming technology and/or virtual reality training environments. Such approaches have been undertaken in recent years and represent an important step towards how we deliver PBT for maximal effect (e.g., The Computer Assisted Rehabilitation ENvironment, or ‘CAREN’ treadmill-based training system [87]).
7. Value of booster training sessions. While PBT can elicit positive gains with minimal training, it has been suggested that this effect could potentially be enhanced with an occasional booster session in the months or years following the initial training protocol [88]. This seems to be a reasonable supplement to any PBT program if time and resources allow.

## 5. Final Thoughts

### Future Directions

In addition to the methodological challenges to overcome when implementing the above ideas, several issues await future study that could increase the efficiency and generalizability of PBT.

1. The optimal ‘training dose’ is unknown. It is encouraging that a single training session can elicit lasting gains in older adults [30], and this may be necessary due to limited time and resources. In such instances, a single, brief training session may be a viable approach. However, if we aim to address how we can optimize PBT, the poor transfer associated with training a single perturbation type makes it clear that there is a trade-off between training time and development of a complete set of balance recovery skills. The optimal training dose to elicit improvement remains an open question.
2. The most common types of perturbations and responses experienced in the real world need to be identified to focus training efforts. Here we need to establish the minimal set of different perturbations and responses that will provide sufficient variability to generalize across the most common causes for falls.
3. Injury mitigation strategies may be an effective means of reducing fall risk [89]. Specifically learning to fall properly may be an effective way to reduce or prevent injury, which is distinct from the idea of preventing the fall altogether.
4. As a final point, we acknowledge that what we have proposed was geared primarily for community-dwelling, healthy older adult populations, which means that suitable adaptations would likely be needed for more vulnerable populations. Future efforts would need to determine how training could best accommodate the unique needs of different clinical groups.

## 6. Conclusions

Although many age-related factors contribute to a loss of balance and subsequent fall risk, once balance is lost, the last resort for fall prevention is a suitable corrective balance reaction. PBT offers a viable solution to target and improve these balance reactions. A noticeable shortcoming with PBT is the lack of transfer from one specific training condition to another. As outlined, a potential solution involves adopting training conditions that emphasize a need for behavioral flexibility (i.e., increasing variability and complexity in the training environment). While such training may take additional time and resources to complete, the benefits of providing vulnerable populations with the necessary skill set to prevent falls in naturalistic settings would outweigh the cost. Indeed, if something as simple as training can reduce the enormous burden falls place on people’s health, finances, and time, then it is imperative that we take steps to maximize the success of these training methods.
